# Transformation Products of Organic Contaminants and Residues—Overview of Current Simulation Methods

**DOI:** 10.3390/molecules24040753

**Published:** 2019-02-19

**Authors:** Lisa Kotthoff, Julia Keller, Dominique Lörchner, Tessema F. Mekonnen, Matthias Koch

**Affiliations:** 1 Department of Analytical Chemistry and Reference Materials, Bundesanstalt für Materialforschung und-prüfung (BAM), Richard-Willstätter-Straße 11, 12489 Berlin, Germany; lisa.kotthoff@bam.de (L.K.); juliakeller19@yahoo.de (J.K.); dominique.loerchner@gmail.com (D.L.); mekonnet@hu-berlin.de (T.F.M.); 2 School of Analytical Sciences Adlershof (SALSA), Humboldt-Universität zu Berlin, Unter den Linden 6, 10099 Berlin, Germany

**Keywords:** transformation product, electrochemistry, photochemistry, Fenton’s reagent, pesticides, pharmaceuticals, brominated flame retardants, mycotoxins

## Abstract

The formation of transformation products (TPs) from contaminants and residues is becoming an increasing focus of scientific community. All organic compounds can form different TPs, thus demonstrating the complexity and interdisciplinarity of this topic. The properties of TPs could stand in relation to the unchanged substance or be more harmful and persistent. To get important information about the generated TPs, methods are needed to simulate natural and manmade transformation processes. Current tools are based on metabolism studies, photochemical methods, electrochemical methods, and Fenton’s reagent. Finally, most transformation processes are based on redox reactions. This review aims to compare these methods for structurally different compounds. The groups of pesticides, pharmaceuticals, brominated flame retardants, and mycotoxins were selected as important residues/contaminants relating to their worldwide occurrence and impact to health, food, and environmental safety issues. Thus, there is an increasing need for investigation of transformation processes and identification of TPs by fast and reliable methods.

## 1. Introduction

**Figure 1:**
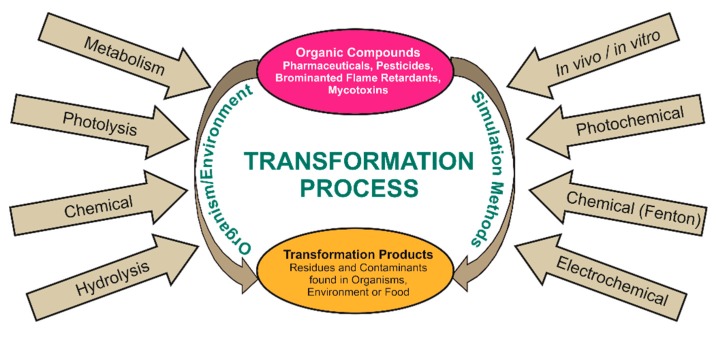
Schematic overview of occurring transformation processes of organic compounds and their corresponding transformation products. On the left side natural transformation processes in organisms and environment are shown, on the right side the most common simulation methods are displayed.

Transformation products (TPs) of organic compounds are structurally diverse intermediates. They are formed by different conversion ways and occur in several matrices worldwide. The knowledge of transformation pathways and identification of TPs is important for health, food, and environmental matters. Metabolism research and investigating effects of TPs is the basis, e.g., drug development or strategies to avoid or reduce harmful TPs in food and environment [1,2]. Figure 1 gives an overview of the occurring transformation processes. TPs are referred as metabolites or degradation products, depending on the origin. Metabolites are formed by biotransformation processes in living organism whereas degradation products are formed by various microbial, biotic, and abiotic processes in the environment.

All these processes cause the formation of TPs from organic compounds. Natural detoxification processes aim to generate easily degradable and harmless TPs. Nevertheless, TPs can occur, which are even more (bio)active or harmful to toxic and as well persistent [2,3,4,5,6]. Consequently, TPs need to be investigated in relation to their formation way and structure. The first investigations on identification of TPs of organic compounds were the scouting of relevant matrices like feces, urine, water, soil, and atmosphere. The identification of TPs in real samples is often challenging due to dilution effects. Moreover, TP identification and studies on their formation processes could be hampered by high matrix complexity especially for TP trace levels. The application of different methods to simulate TPs allow the above mentioned issues and moreover these methods are faster and cheaper [7]. Another benefit is the production of TPs in higher amounts with high purity [2]. This offers the possibility for risk assessment purposes, like toxicity tests or the conceivable use of the isolated TPs as reference material. All transformation processes are based on simple chemical reactions like oxidation, reduction, hydroxylation, hydrolysis, cleavage, and dealkylation [2,8]. These reactions correspond to the phase I reactions of the metabolism and are also reactions that take place in the environment. Further typical phase II reactions like conjugation or methylation are involved in the metabolism process.

**Figure 2:**
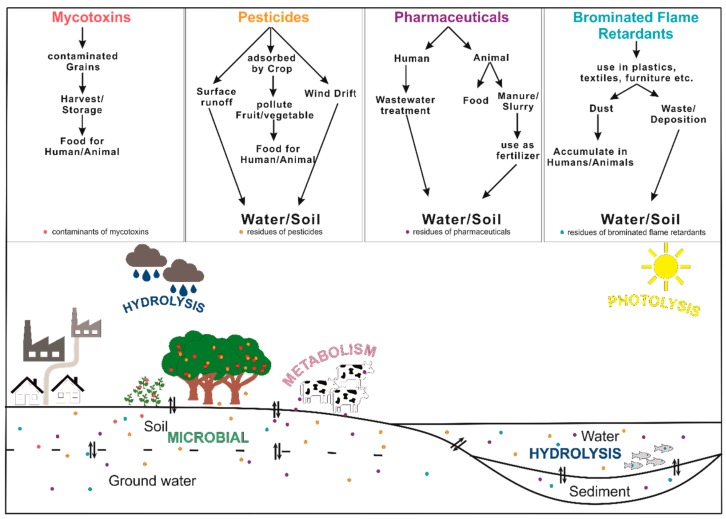
Contaminants and residues of mycotoxins, pesticides, pharmaceuticals, and brominated flame retardants can enter different environmental matters including water, sediment, soil, or groundwater by different pathways. The main transformation processes of hydrolysis, photolysis, metabolism and microbial activity lead to a broad spectrum of TPs that are widely distributed.

After metabolization or degradation of the parent compounds, TPs (or even the unmetabolized compounds) are excreted and can enter environmental systems (see Figure 2) like water, sediment, soil, and biota, where they could have an impact on these matrices [3,5,6,9]. The environmental surroundings and characteristics (water, light, irradiation, temperature, pH, humidity, atmosphere, and soil [4]) are important for further transformation pathway, including generation of new TPs. On the other hand, industrial processes such as waste water treatment, there are different processes used to eliminate residues or contaminants from the water cycle. Relevant organic compounds are manifold and there are substances found in all areas. Most prominent residues are pharmaceuticals (drugs), pesticides and personal care products. But many more chemical compounds contribute to the environment like brominated flame retardants (BFRs), industrial additives, and surfactants [2,3,6,8]. Next to the artificial compounds occurring in the environment, natural contaminants like mycotoxins (secondary metabolites of fungi), or alkaloids (produced by organisms like bacteria, fungi, plants, and animals) have an impact to environmental and food samples [10,11]. Pharmaceuticals and drugs are used worldwide in a high amount, BFRs have persistent and bioaccumulative properties and mycotoxins show a high toxicity even in low concentrations. Both synthetic and natural compounds are often occurring in foodstuff (milk, eggs, seafood, fish, wheat, etc.) and their consumption is nearly inevitable. Therefore, the investigation of TPs regarding their formation processes, structural identification and occurrence in different foods, and environmental matrices is of increasing relevance for industry, the scientific community, and regulatory bodies as well.

There are some reviews dealing with the analysis and characterization of residues and contaminants including TPs [3,4,5]. However, literature on the trends and applications of simulation methods is rather limited. Hence, the aim of this review is to describe current developments and trends of methods to simulate TPs of compounds formed in natural and manmade processes. The simulation methods could be categorized in photochemical-based, electrochemical-based and Fenton-based methods. Also, the well-established in vivo and in vitro methods for investigation of metabolic processes are addressed. Due to the commonly increased polarity of compounds during transformation, TP identification is preferably done by liquid chromatography coupled to (high-resolution) mass spectrometry, LC-(HR)MS.

## 2. Methods for Transformation and Analysis

### 2.1. Metabolism Methods

In vitro and in vivo experimental models are useful approaches to identify major metabolism pathways [1,7]. The metabolism (of living organism) of exogenous compounds is also known as biotransformation processes and takes place mainly in the liver. The biotransformation process is divided into two phases, whereby the aim is to increase the polarity of the compound to allow the elimination from the organism. In phase I mainly oxidation, reduction or hydrolysis occur and they are catalyzed by cytochrome P450 (CYP450) enzymes [12,13,14]. The phase I metabolites can be excreted or a further conjugation reaction (phase II) with biomolecules such as glucuronic acid or glutathione [7,15,16] takes place. There are a lot of different models for tests available with a different complexity, starting from test tube experiments to cell cultures, animals, healthy human subjects, and clinical trials [17]. There are different in vitro human liver models developed, including supersomes, microsomes, S9-fraction, cell lines, transgenic cell lines, primary hepatocytes, liver slices, and perfused liver [15]. For these methods, only a low concentrated test substance can be used and, consequently, less amounts of metabolites are generated. The biotransformation process is initiated by a single electron transfer or hydrogen atom transfer involving an iron–oxygen complex [18]. Major CYP450 catalyzed reactions are oxidations like carbon hydroxylation, heteroatom dealkylation, heteroatom oxygenation, bond oxidation, hydrocarbon desaturation, dehalogenation, and epoxidation [1,16,19].

Another commonly used metabolism method is the incubation of target compounds with human (HLM) or rat (RLM) liver microsomes. After incubation, the metabolites are isolated and analyzed with chromatographic techniques coupled to MS or DAD/FLD detection [16].

### 2.2. Electrochemical-Based Methods

Electrochemistry (EC) and electroanalytical techniques offer the opportunity to study the redox properties of chemical compounds [7,16,20,21]. The electrochemical transformation can proceed direct or indirect. By direct electrochemical transformation the product is formed by an electron transfer between electrode and compound. Otherwise, reactants or mediators are electrochemically generated and initiate the transformation of the compound of interest [21]. There are different electroanalytical techniques available like potentiometry (the difference in the potentials of two electrodes is measured as voltage), coulometry (a charge is determined), amperometry (a current is measured), and voltammetry (a current is measured while changing the potential of an electrode). Electroanalytical experiments can be performed easily using a typical electrochemical cell consisting of a two- or three-electrodes and a potentiostat. The electrochemical oxidation, which occurs by applying a potential, lead to comparable reactions like phase I reactions of the biotransformation process. Ultimately, EC mimics reactions initiated by a single-electron transfer like *N*-dealkylation, *S*- and *P*-oxidation, alcohol oxidation, dehydrogenation reactions, hydroxylation of activated aromatic compounds, and dehalogenation [18,20,22].

Advantages of EC are that results are quickly generated, and the generation takes place matrices free. The EC-reactor equipped with a thin-layer flow through cell has the advantage of an online coupling to different devices like LC-DAD/FLD, LC-MS, or mass spectrometric detectors [16,20,23]. The generation of TPs and identification is possible with one system, thereby also short-lived TPs can be identified. Next to the simulation of phase I reactions (oxidations or reductions) EC/MS could be used to simulate phase II reactions. For this purpose, reactive species generated from EC are trapped by selected biomolecules such as glucuronic acid or glutathione before entering to MS techniques. 

### 2.3. Photochemical-Based Methods

Photolysis is generally a light/photoinduced cleavage of a chemical bond of a molecule. Photolytic processes occur in the atmosphere and at the surface of water and soil [2]. The transformation process is initiated by the absorption of light. Direct photolysis happens when the compound of interest absorbs light and then a transformation process is started, which breaks up bonds. Indirect photolysis occurs, when an additional second molecule, naturally occurring (like humic acid or nitrate), absorbs the light and generate strong reactive oxygen species (singlet oxygen, hydroxyl radicals, or alkyl peroxyl radicals). These radicals initiate the transformation process of the target compound [4,24]. In general, the rate of photolysis is dependent on numerous chemical and environmental factors, including the light absorption properties, reactivity of the compound and the intensity of solar irradiation. Substances with double bonds or hetero atoms can absorb light between 200 nm and 800 nm depending on the present structural properties [9].

The natural photolytic processes are simulated under laboratory conditions to investigate the influence of different parameters on the photolysis and the occurring TPs. For the photolysis experiments, the compounds of interest are irradiated for a determined time with light of certain wavelength. Functional groups of the compounds absorb light of the UV area between 100 nm and 400 nm. The UV light can be divided into four groups: (i) VUV light (100–200 nm), (ii) UV-C (200–280 nm), (iii) UV-B (280–320 nm), and (iv) UV-A (320–400 nm). There are different light sources available, divided into mono- or polychromatic. With polychromatic light sources a defined wavelength range is used for the irradiation and the range is selected by filters. Sunlight is also an irradiation source and can be simulated by polychromatic light sources like a Xe-lamp. In environmental areas sunlight is responsible for the occurring photolysis. Sunlight did not contain UV-C light, but for photolysis experiments this is used, because of its high energy and this light can also simulate processes of particle absorption in the atmosphere. Mercury-vapor lamps and low-pressure mercury-vapor lamps mainly emit light at 254 nm and are used as monochromatic light sources to investigate the influence of UV-C light. Further simulation methods are photocatalytic experiments. The most common catalyst is TiO_2_ [25]. The catalyst molecule absorbs the electromagnetic irradiation and the excited TiO_2_ produce oxidative or reductive radicals (e.g., hydroxy radicals). These radicals take part in the transformation process of the compound. Simulated reactions are such as hydroxylation, dehydrogenation, and/or hetero atom dealkylation [26].

With ozonation and chlorination highly reactive radicals are built, which start the transformation process. For that purpose, photooxidants (e.g., O_3_ and Cl_2_) are added to the reaction solution and then irradiated with UV light [27,28]. These methods are often used in waste water treatment, where the aim is to eliminate organic substances. Primarily, advanced oxidation processes or disinfection (via chlorination, ozonation, or UV irradiation) are commonly used, but the possibility of generating new TPs by these applications is also given [8,27].

### 2.4. Fenton-Based Methods

Fenton’s reagent contains hydrogen peroxide and iron(II)-ions [28]. It is a chemical process, where the iron salt acts as a catalyst by the decomposition of hydrogen peroxide. Due to the decomposition of hydrogen peroxide are hydroxyl radicals generated. These hydroxyl radicals are highly reactive and initiate the transformation process of the compound of interest [28]. The classical Fenton’s reagent is used in an aqueous milieu. Fenton-like reactions use other peroxides and/or iron(III)-salts. Next to the classical Fenton reaction also combinations between electrochemistry or photolytic assisted Fenton reactions are used [26,28,29,30,31]. Here, the Fenton’s reagent is produced either electrochemically (hydrogen peroxide is generated simultaneously through the impact of an applying potential) or photochemically (iron complexes absorb the UV light). The transformation process is initiated by the generated hydroxyl radicals. 

### 2.5. Analytical Techniques for TP Identification

Analytical techniques are indispensable for TP identification. Today, the most commonly used analytical techniques for TP identification studies are based on chromatographic separation, preferably achieved by gas [32,33,34] or liquid chromatography with high separation performance [3,5,34]. Liquid chromatography systems are powerful tools for fast, accurate, sensitive, and selective determination of trace amounts of investigated samples. The further development of high-performance liquid chromatography (HPLC) to ultrahigh performance liquid chromatography (UPLC) leads to shorter analysis time, more/higher sensitivity and improved repeatability. These advantages contribute to a faster and more reliable identification of the TPs [35,36].

Mass spectrometry gained increasing importance for detection in recent years because of strongly improved technologies and wide applicability. Before the MS detection the separated compounds have to be ionized, which is preferably achieved by electrospray ionization (ESI) or atmospheric pressure chemical ionization (APCI) [37,38]. Whereas the ESI technique is mainly applied for (medium-) polar substances, APCI is rather used for nonpolar substances. Due to a usual polarity increase during transformation from parent substance to TPs, ESI is commonly used for TP analysis [37,38].

Different mass analyzing techniques are known: Single and triple quadrupole (QqQ), time-of flight (TOF), ion trap (IT), Fourier transform ion cyclotron resonance (FT-ICR), and Orbitrap [2,37]. Besides the classical use for quantitative determination of targeted analytes with MS, suspected and unknown TPs could be identified. The high-resolution (HR) mode of FT-ICR, Orbitrap MS, and TOF-MS enables the measurement of the high accurate molecular mass of TPs, which allows the determination of an empirical formula [2,3,39]. Among the HRMS systems, the FT-ICR reveals the highest performance in ultrahigh-resolution and mass measurement accuracy [40,41]. Also, hybrid configurations are well established like quadrupole-time-of-flight (Q/TOF), quadrupole-ion trap (Q/IT), linear trap quadrupole Orbitrap (LTQ-Orbitrap), or linear trap quadrupole FT-ICR (LTQ FT-ICR). The coupling enables mass spectra of multiple product ions with accurate mass measurements [2,37]. The MS-based approaches of suspected or nontargeted-screenings are also useful for the identification of unknown TPs [2,42,43,44,45]. Other available orthogonal techniques next to MS for unambiguous structural identification like nuclear magnetic resonance spectroscopy (NMR) [2,46] or X-ray crystallography (XRC) analysis are also powerful tools for structural identification of TPs. These techniques are often used in addition to chromatographic and mass spectrometric methods. 

## 3. Transformation Processes of Selected Compound Classes

### 3.1. Pesticides

For many decades, pesticides are one of the widely regulated residues all over the world. However, due to their direct contacts with terrestrial animals and plants, they continue as a primary concern for food safety. Furthermore, the parent compounds degrade to many TPs by either of metabolism in living organisms (plants, animals, and microbes) or other environmental breakdown processes (e.g., thermal or UV degradation). Nevertheless, several regulations do not require the inclusion of maximum residual levels of TPs in food and environmental risk assessments. Hence, little is known about TPs and their formation mechanisms regardless of their frequent occurrence in food products [47,48], water bodies [49], and animals [50,51]. One of the many reasons is the lack of authentic standards of TPs. For instance, Bauer and coworkers detected many TPs of azoxystrobin, difenoconazole, and thiacloprid in vegetables [47]. However, they were able to confirm only three TPs of thiacloprid (thiacloprid-amide, thiacloprid-O-analogue and 4-hydroxythiacloprid) due to lack of commercially available standards. Zhang et al. have been investigated *N*-dealkylation, nucleophilic substitution of chlorine and many glucuronidation metabolites of forchlorfenuron (a total of 17 TPs) in kiwifruit by untargeted time-of-flight-MS approach [52]. Often, suspected and nontargeted MS based metabolomic approaches, similar to Zhang et al.’s [52], Du et al.’s [44], Wang et al.’s [45], or Choudhury’s [53] studies, have been used to overcome the rare availability of authentic standards. However, reliable confirmation by NMR or authentic standard is desirable yet. Beside this, matrixes complexity, instability (immediate degradation or conjugation) and concentration variation in a specific matrix are always an analytical challenge for TPs investigation in real samples [44].

On the other hand, a variety of simulation methods with the aim to predict or to synthesize TPs of pesticides have been developed successfully. Like pharmaceuticals, investigation of the fate of pesticides in living organisms using the conventional in vitro and in vivo methods have been used as a common practice. Obviously, most products formed by these methods are metabolites and use stereoselective enzymes. On the contrary, in vitro and in vivo methods are hampered by biological materials cost, ethical considerations, matrixes complexity, and time of analysis [54]. To overcome some of these challenges, the so called “organ-on-a-chips” methods are recent significant developments based on microfluidics, which enable prediction of biotransformation products without biological matrices [55]. On the other hand, transformation in abiotic systems is even more difficult to predict since the parent pesticide could expose to multistress factors. Nevertheless, different laboratory scale simulation mechanisms are commonly used for decades [56]. For example, photolysis (UV and visible light), photochemical, Fenton, and advanced oxidation systems (e.g., O_3_, O_3_/H_2_O_2_, O_3_/UV, sonolysis, permanganate, and electrochemical treatments) were used widely to study either kinetics, mechanisms, or type of TPs of pesticides [51,57,58,59,60]. Negreira et al. [51] oxidized ethoxyquin using H_2_O_2_/2,2-diphenyl-1-picrylhydrazyl and predict its *N*-oxide, hydroxylation, *O*-dealkylation, dimerization, epoxidation, and quinone TPs. Furthermore, dimerization, *N*-dealkylation, and cyclization TPs of the herbicide bentazol have been reported by Berberidou et al. [57] using TiO_2_ and ZnO photocatalytic simulation methods. Some, but not all, TPs could be simulated by such laboratory scale methods. However, one of the great challenges of such methods is how well the transformation mechanisms and type of TPs represent the real abiotic transformation processes. Many advanced oxidation methods use strong oxidizing agents that lead to multiple hydroxylations and radical reactions or complete mineralization of the target compound. Beside this, photolysis and photocatalytic methods are more prone to a nucleophilic substitution of halogens and polymerization [60]. Not only the efficiency of simulation methods but also the effect of produced TPs should be addressed. For instance, chemical processes like chlorination could product carcinogenic disinfectant by-products [3,56]. Recent efforts towards the investigation of TPs pesticides in different matrices, prediction methods and main transformation mechanisms have been summarized in Table 1. 

Beside this, EC coupled to LC-MS gets considerable attention to investigate biotransformation processes of pesticides. In 2009, Lohmann et al. [61] used EC/LC-MS to predict hydroxylation, the nucleophilic substitution of chloride, and amination of boscalid. In this work, they are able to predict phase II conjugative metabolites (with glutathione) too. Typically, a compound with an appropriate organic modifier and electrolyte solution is infused into an electrochemical cell consisting of the working electrode coupled to online MS or LC-MS. Two works from Mekonnen et al. [62,63] have been sought biotransformation processes of a fungicide and an insecticide using EC/MS in comparison to commercially available rat and human liver microsomes. Several TPs of chlorpyrifos and fluopyram via hydroxylation, *P*-oxidation, dealkylation (*O*- & *N*-), and nucleophilic substitution of halogens were identified by LC-MS/MS and HRMS. Many biotransformation reaction mechanisms (Table 1) could be easily simulated by EC based redox reactions. Additionally, synthesis time, mechanism elucidation and automation (online synthesis, separation and identification of TPs) could be significantly improved by EC coupled to MS. Recently, a review by Mohle et al. [64] discuss the possible scale-up of EC to synthesize value added organic products. For example, synthesis of benzaldehyde from dimethoxylation of 4-tert-butyltoluene and reduction of acrylonitrile to adipontrile for polyamide production are industrial scale processes using organocatalytic electrochemistry. Instead of using reagents, applying electricity for chemical production is a green approach (since no generated wastes) and economically valuable. Furthermore, EC uses environmental friendly reagents like water as a source of hydrogen or hydroxyl, molecular oxygen for oxidation or CO_2_ for carboxylation processes [64]. Inline of this, Mekonnen et al. [48] have been synthesized reference standards of TPs (nonfractionated) using BDD (boron-doped diamond) electrode for unbiased authentication of chlorpyrifos metabolites in foodstuff. Meanwhile, the EC/MS approach has limitations on stereoselectivity and representation of all enzymatic processes [54]. However, this method could be an advantage in future to synthesize both metabolites and abiotic TP reference standards at the same time [48].

In summary, pesticides’ transformation products investigation in foodstuff and environmental samples are very rare and limited only to TPs whose standards are commercially available. On the other hand, there are no common prediction methods for both biotic or abiotic transformation processes of pesticides. Despite many instrumental and simulation methods developed so far, quantitation and unbiased identification of TPs of pesticides in food and environmental samples are basic analytical challenges yet.

### 3.2. Pharmaceuticals/Drugs

The use of medication has a major role in people’s and animal’s health. Pharmaceuticals are not only used against human infections, but also intensively in the food industry, veterinary medicine, and agriculture [1]. In drug discovery and development the fate of the drug after disposition in the body is investigated, this involves the absorption, distribution, metabolism, and excretion (ADME) [69]. Most drugs are metabolized in the liver, where CYP450 enzymes are highly concentrated. For this reason, HLM or RLM can be used for simulation of phase I biotransformation reactions [70]. Through the excretion unmetabolized drugs (parent compounds) and their metabolites reach the environment [2,27].

Today in the TPs research of drugs there are two different areas, where simulation methods are used as mentioned in the introduction part. First, in drug research and development, TPs are investigated to determine generated metabolites and to understand the possible biotransformation pathway inside the body. Also, the determination of the excreted residues is important [16]. Since the last decades in environmental analytics, especially the water analysis and water treatment, TPs were investigated [4,5]. Descripted above, TPs reach the water cycle and are detected. Through some treatment processes new TPs could be formed [71]. Here photolysis and photocatalytic processes are significant. Often the focus of these studies is the elimination of drugs from wastewater including kinetic studies and not the identification of TPs, but sometimes the degradation pathway is described.

In Table 2 some chosen studies are given, where different simulation techniques are compared. Szultka-Mlynska et al. [69,72] used EC/LC-MS for the investigation of TPs of a various number of chemotherapeutic and cardiovascular drugs. They could show that for the chemotherapeutic drugs some of the EC-generated and identified TPs were also identified in RLM tests. The TPs of the cardiovascular drugs were compared to in vivo samples (urine and plasma). It turned out that some EC-simulated TPs were also available in the in vivo samples. Further to show continuation, three different simulation methods (photocatalysis (TiO_2_), EC-Fenton, and EC-reactor) were used and the resulting TPs are compared to HLM-identified TPs by Ruokolainen et al. [26]. They tested buspirone, promazine, testosterone, and 7-ethoxycoumarin. A different number of TPs could be simulated for all four compounds, and the number was higher in contrast to HLM identified metabolites. TPs of testosterone and 7-ethoxycoumarin could not be generated by EC. For buspirone could two (hydroxybuspirone and 5-hydroxybuspirone) of three metabolites (plus busprione *N*-oxide) be found with photocatalysis, but not with the other used methods. For promazine (TPs: *N*-desmethylpromazine, promazine sulfoxide, and 3-hydroxypromazine), two of three metabolites (*N*-desmethylpromazine and promazine sulfoxide) were found with EC-reactor (basic pH conditions) and one of them (promazine sulfoxide) also with EC-reactor (acidic pH conditions) and EC-Fenton. The third metabolite 3-hydroxypromazine was found by photocatalysis. For testosterone, both TPs are found with EC-Fenton and one also with photocatalysis. For 7-ethoxycoumarin one TP was found with photocatalysis and EC-Fenton. As a result, photocatalysis simulates better HLM-TPs than the EC-based methods. 

Furthermore, Serna-Galvis et al. investigated the β-lactam antibiotic oxacillin with photocatalysis (TiO_2_), sonochemistry, EC-oxidation (Ti/IrO_2_), and photo-Fenton (Table 2) [73]. In total, there were nine different TPs (TP1–TP9) detected. The EC-oxidation led to three different TPs (TP1: breakdown of the central secondary amide, TP2: isomerization, and TP3: opening β-lactam ring, *S*-oxidation), which no other methods simulate. The photo-Fenton generated the other five TPs (TP4: opening β-lactam ring, TP5 breakdown of the central secondary amide, TP6: *S*-oxidation, TP7: aromatic hydroxylation, TP8: opening β-lactam ring, and TP9: decarboxylation), three of them are also observed with photocatalysis (TP5, TP7, and TP8) and sonochemistry (TP4, TP5, and TP6). Another study by Lecours et al. compared electrolysis, EC-Fenton, and EC-reactor methods for the simulation of TPs of Trimethoprim [18]. The α-OH-Trimethoprim as TP was simulated with all three methods and is described in the literature as a metabolite of rat models. The electrolysis did not produce further TPs. The other both methods produced more, but less selective TPs. Two further TPs were obtained with the EC-reactor and four more TPs with the EC-Fenton. Most of the identified TPs were known from earlier studies with for example photolysis/photocatalysis experiments or photo-Fenton investigations. Also listed in Table 2 is that Gawlik et al. [74] investigated the TPs of the antidepressants moclobemide and toloxatone with photocatalysis (TiO_2_) and HLM incubation tests. The seven TPs of moclobemide of the HLM tests were all simulated with the photocatalysis experiment. The same results are found for toloxatone, here are four identified TPs simulated with the photocatalysis, but three additionally TPs were identified. Another study of this group dealt with HLM-incubations, TiO_2_ photocatalysis, and ZnO-photocatalysis of clozapine [70]. The HLM-study resulted in seven TPs. All TPs could be observed in the ZnO photocatalysis. Among all TPs produced by ZnO and TiO_2_, only a further *N*-oxidation TP was not generated by HLM. On the other hand, TiO_2_ could simulate six if the seven HLM metabolites. The HLM metabolism (seven TPs) could be simulated for this compound completely with the ZnO photocatalysis method. Besides studies comparing different simulation techniques also several investigations with one method are available. The photolytic and photocatalytic experiments are often evaluated in relation to degradation kinetics and toxicity of TPs. This is important for the environmental studies, to predict the impact of the TPs on the general ecosystem. 

Depending on the investigation objective, different methods are used. Electrochemistry is commonly used for the simulation of TPs occurring during the metabolism of organism/animal and human. To predict and investigate TPs occurring in the environment, photolytic, and photocatalytic experiments are widely used. However, there are also a few studies where electrochemistry methods are used in the environmental science [18] and photolytic/photocatalytic experiments to simulate in vivo or in vitro metabolites [70,74]. Typical metabolic redox reactions can be mimicked with most of the above described simulation methods. Until today, the one-to-one simulation of metabolic reactions is unfeasible with only one simulation method. The metabolism is a complex procedure and some structural properties of the drug of interest are significant. The simulation methods often produce unspecific reactions, especially if they are radical driven. For the investigation of metabolically-occurring TPs at least microsome tests are necessary as a reference method. For the investigation of environmental relevant TPs environmental samples like soil or water are analyzed, where by nontargeted analytic TPs are found. 

All named simulation methods are important to generate TPs of drugs and all methods show the possibility to generate relevant TPs due to comparisons to metabolism studies or screenings of environmental samples. The fate of the parent compound and their TPs could be described more detailed using different methods. Through the high usage of drugs in daily living life and the high number of available drugs, the number of TPs of drugs is probably far higher than assumed. These TPs may be harmful to the environment and living organisms and it is crucial to examine TPs and to produce reference standards and materials of emerging/novel TPs. 

### 3.3. Brominated Flame Retardants

Brominated flame retardants (BFRs) are used in a wide range of polymer materials to reduce the flammability due to lower costs and high efficacy in comparison to inorganic and phosphorous or nitrogen containing organic flame retardants [85,86]. However, these structural diverse compounds have the disadvantage of persistent and bioaccumulative potential, which is an issue of concern for human, animal and the environment [85]. Furthermore, so-called additive BFRs, which are implemented in the polymer material, may migrate in the environment and due to the global distribution high levels of BFRs were identified worldwide [87,88]. Therefore, the occurrence and fate of brominated flame retardants in humans and in the environment are topics of increasing concern. In recent years, numerous studies about their global transport, UV degradation, bioaccumulation, and toxicity were performed to assess their environmental fate. Toxicological chronic effects and endocrine disrupting activity present the important issue [89,90] for further investigations relating to degradation or transformation behavior as well as the identification of TPs of these compounds. 

Studies about electrochemistry coupled to mass spectrometry (EC/MS) to simulate biotransformation or environmental processes are less described relating to BFRs. The present studies focused mainly on degradation processes and identification of TPs by LC-(HR)MS or GC-MS. A proposal of the degradation mechanism is not described. Nevertheless, they give first insights in the electrochemical/photoelectrochemical behavior of some BFRs, which can predict the behavior in human and in the environment. Heberle et al. investigated the photo electrolysis of 2,4,6-Tribromophenol (TBP), which results in a mineralization as well as debromination reactions [91]. In comparison, Markova et al. reveals a monomeric and dimeric alkoxy derivatives of TBP by using a Pt electrode but a mineralization of TBP was not observed [92]. The electrochemical reduction of hexabromocyclododecane (HBCD) at carbon and silver cathodes in dimethylformamide occurs mainly in a cleavage of the C–Br bond and in the formation of subsequent degradation products with different degradation routes [93]. Xu et al. investigated the oxidation and reduction potential of 4-bromophenol using a divided cell whereby on Pd-Fe/graphene catalytic cathodes reduction takes place with adsorbing hydrogen and on Ti/IrO_2_/RiO_2_ anode where oxidation reaction is performed. EC products were identified by LC–MS or LC-IC and mainly debrominated products induced the formation of phenol. Additionally, hydroxylation to hydroquinone, fumaric acid, succinic acid, aromatic, and carboxylic acid intermediates were observed [94]. A new approach for electrochemical oxidation of brominated phenol with carbon fiber brush electrode was reported by Skopalova et al. Eleven new oligomeric products including quinones and biphenoquinones were identified by LC–MS and GC–MS as well as characterized by X-ray crystallography [95]. Overall, the obtained TPs are mainly debromination and hydroxylation reaction products by electrochemical oxidation and/or reduction and reveals that the new products are more polar than the initial compounds. Hence, a possible distribution in the environment, humans and animals may prefer. 

Investigations on UV and simulated sunlight irradiation are easy and fast methods to predict the environmental behavior of BFRs. In comparison to electrochemical studies the focus is on degradation pathway, kinetics and characterization of the photolysis products. Wang et al. investigated the photolysis of ten lower brominated diphenyls (BDE) congeners and suggested that the ortho-bromine position is prerequisite for converting these polybrominated biphenyl ethers (PBDEs) into polybrominated dibenzofurans (PBDFs) [96]. Wang et al. also examined three BDE-congeners (-29, -25, and -21) under UV light to evaluate the degradation mechanism and pathways. Debromination reactions play a significant role whereby the debromination position are totally different for these BDEs. They find out that the debromination takes place at each position and therefore, the debromination pathway cannot be summarize. PBDEs with ortho-bromine substituent can generate lower PBD furans by UV light [97]. Furthermore, BDE-47 was investigated with Fe(III) and/or fluvic acid in lake water by Zhao et al. and identified hydroxylation products under simulated sunlight. 6-OH-47 and 2′-OH-68 were the main photolysis products and the concentration of these products do not increase with an increase of photoreactive components [98]. The photolysis of tetrabromobisphenol A (TBBPA) in water under simulated sunlight was reported by Wang et al.; 2,6-dibromophenol and two isopropyl phenol derivates are the main photooxidation products, but also reductive debromination products as well as the formation of hydroxyl-TBBPA could be observed, identified by LC–MS and characterized by FT-ICR-MS, NMR, ESR, and IRMS [99]. Bao et al. investigated the photochemical behavior of TBBPA also under simulated sunlight. TBBPA was degraded by a kinetic pseudo-first order to debrominated products and the cleavage of C-C bond [100]. The photodegradation of 1,3,5-Tris(2,3-dibromopropyl)-1,3,5-triazine-2,4,6-trione (TDBP-TAZTO) under UV light was reported by Liang et al. TDBP-TAZTO was decomposed after 120 min but they give no information about transformation products as well as the degradation pathway [101]. Zhang et al. described the photochemical transformation of five novel BFRs: allyl-2,4,6-tribromophenol ether; 2-bromoallyl-2,4,6-tribromophenyl ether; 2,3-dibromopropyl-2,4,6-tribromophenyl ether (DBTE); 1,2-bis(2,4,6-tribromophenoxy)ethane (BTBPE); and 2,4,6-tris(2,4,6-tribromophenoxy)-1,3,5-triazine under simulated sunlight. All BFRs were photochemically degraded and the environmental half-lives were calculated. Only for DBTE and BTBPE degradation pathways were proposed and the main pathway includes debrominations for both compounds as well as ether bond cleavage for BTBPE [102].

These simulation techniques were often compared to commonly in vitro experiments in biological systems. In vitro experiments with human liver microsomes to evaluate the biotransformation 1,2-dibromo-4-(1,2-dibromoethyl)cyclohexane was reported by Nguyen et al. Five metabolites were identified by LC-HRMS measurements and the main reactions include the mono- and dihydroxylation, alpha-oxidation, and debromination [103]. Su et al. described the biotransformation of tetradecabromo-1,4-diphenoxybenzene and its photolysis products (UV and sunlight) using herring gull and rat liver microsomes This BFR was slowly metabolized, but the metabolization of the formed photolysis products were proceed faster [104]. PBDEs were well investigated relating to environmental behavior, but in vitro experiments with hydroxylation products are less. However, Li et al. studied OH-PBDEs with pig liver microsomes and find out that these compounds were more metabolized as PBDEs. The biotransformation decreased with an increase of the number of bromine atoms and the dominant pathway was the cleavage of the diphenyl ether bond. Hydroxylation and debromination reactions are less observed [105]. Similarly, Zheng et al. investigated the in vitro metabolism of BDE-47, BDE-99, and HBCD using chicken liver microsomes. Six hydroxylated tetra-BDEs metabolites could be identified by LC–MS with authentic standards of BDE-47, three hydroxylated penta-BDEs for BDE-99 and four monohydroxylated as well as more than five dihydroxylated metabolites for HBCD [106].

Conclusively, hydroxylation and debromination reactions are the main degradation pathways of BFRs using these different techniques. The comparison of all techniques for only one compound is not possible until now. Furthermore, studies on degradation in real samples are less and there is a strong need for further investigations on this field. However, a prediction of the environmental behavior of these compounds is possible. The investigation of the fate and occurrence of some BFRs in humans and animals is more difficult due to the low solubility in water. Therefore, toxicity tests and in vitro experiments are indispensable for this kind of chemicals to evaluate the fate and occurrence in human and animals.

### 3.4. Mycotoxins

Mycotoxins are secondary metabolites of filamentous fungi occurring worldwide in food and feed and have been found to cause severe diseases in humans and livestock. Their consumption is nearly unavoidable due to their ubiquitous presence in food and feed and maximum levels are set to ensure food safety. Elucidation of metabolic pathways of various mycotoxins is crucial to understand their toxicity. As a result, innovative techniques are needed to allow faster achievement of reliable data concerning mycotoxins and their TPs. Former studies concentrated on the degradation of mycotoxins to decontaminate food and feed. Meca et al. [107] investigated the interaction between the mycotoxin beauvericin (BEA) and nine yeast strains of *Saccharomyces cerevisiae* to biologically degrade the mycotoxin under aerobic conditions in a liquid medium of potato dextrose broth (PDB). Using LC–MS/MS they found that BEA was reduced meanly by the yeast strains in PDB by 86.2% and by 71.7%. in a food/feed system composed of corn flour. One degradation product was found and characterized by its MS/MS fragmentation pattern, which revealed a loss of the amino acid *N*-phenylalanine and two water molecules. Chemical reduction of BEA was also investigated using allyl isothiocyanate (AITC), a natural ingredient of mustards [108]. Measurements by linear ion trap mass spectrometry showed the reduction of BEA ranged from 20 to 100% in a time-dependent fashion. Two reaction products have been detected corresponding to BEA conjugates with AITC molecules. A study conducted by Bordin et al. [109] investigated the reduction of the mycotestrogen zearalenone (ZEA) and α-zearalenol (ZEL) on a solution model using AITC. Mycotoxin reductions were dose-dependent and conjugates of ZEA with AITC as well as ZEL with AITC were described. 

Further degradation methods were the irradiation with UV light as described by Schmidt-Heydt et al. [110]. In this study ochratoxin A and B (OTA and OTB) and citrinin (CIT) are degraded by blue light. CIT is completely degraded, whereas OTA and OTB did no longer contain phenylalanine, as confirmed by LC–MS/MS. The analysis revealed that in all cases modifications or cleavages at the phenylalanine moiety occurred. Also, with pulse light technology several mycotoxins were degraded or inactivated as described by Moreau et al. [111]. In this study the mycotoxins ZEA, deoxynivalenol, aflatoxin B_1_, and ochratoxin were tested. The authors of the study focused on decreased toxicity of the degradation products but did not investigate possible reaction products. Besides several studies dealing with the degradation of mycotoxins using irradiation with light, microbial, chemical, and biological detoxification so far (please see reviews from Kabak et al. 2006 and McCormick 2013 [112,113]), only a handful of studies concentrated on the investigation of TPs of mycotoxins. 

Often detoxification processes of organisms like human and higher animals during biotransformation lead to new toxic compounds. Investigation of metabolic processes is crucial to get an understanding of toxicity modes of action. Currently, reference substances are scarce to even non-existent and impede the investigations of potentially harmful TPs. Since electrochemistry coupled online to different mass spectrometric devices is well established in pharmaceutical research electrochemistry in Mycotoxin Res. was used as a pure detection method. The combination of square wave voltammetry and the standard additions method was proposed as an alternative analytical method for a rapid screening of the mycotoxin patulin and 5-hydroxymethylfurfural in apple juices as described in Chanique et al.’s work [114]. Calcutt et al. [115] showed a direct correlation between the electrochemistry of OTA and 4-chlorophenol and for both compounds, subsequent scans revealed the occurrence of hydroquinone/benzoquinone couples, which were produced from the oxidation of the parent *para*-chlorophenol moiety. Besides cyclic voltammetry, other electrochemical detection methods including immunosensing strategies and affinity biosensor are often used and have been reviewed recently (Vidal et al. [116] and Catanante et al. [117]). 

However, the first description of EC used as a purely instrumental setup to simulate biotransformation processes was performed with alternariol (AOH) and alternariol methyl ether (AME). Simon et al. [118] investigated these two representatives of the Alternaria toxins concerning their oxidative phase I metabolites using EC coupled to HR-ESI-MS and EC/LC–MS equipped with a boron-doped diamond working electrode in an amperometric thin-layer cell. By applying continuous increasing potentials from 0 to 2500 mV vs. Pd/H_2_, several reaction products derived from AOH and AME were detected and described. Mostly hydroxylation and adduct formation with methanol from the solvent was observed. Results obtained from EC were also compared to in vitro assays with HLM. The incubation with HLMs led to the formation of less products compared to EC, but monohydroxylated species were comparable. Simon et al. concluded that the oxidation by EC could be considered as very useful to generate in vitro metabolites of mycotoxins. 

Two studies from Keller et al. [119,120] recently investigated the electrochemical oxidation of CIT, dihydroergocristin (DHEC), and ZEA and compared the results to those obtained from microsomal incubations with RLM and HLM. Several monohydroxylated ZEA, DHEC, and CIT species have been observed in both the electrochemical and enzymatic test systems. The electrochemical setup consisted of an electrochemical flow-through cell equipped with a glassy carbon and a boron-doped diamond working electrode coupled online to a single quad MS system. Identification of the species was performed by HRMS and LC-MS/MS measurements. Both studies concluded that EC could not fully simulate the biotransformation processes of the three tested mycotoxins, but could lead to interesting and new reaction products like dimers (in case of ZEA). As well tested for their simulation potential was the Fenton-like reaction, also described in both studies. The Fenton-like reaction is driven by its two components—iron(III) and hydrogen peroxide—and is often used to destruct hazardous organic pollutants in wastewater by nonselective oxidation. In both studies, the Fenton-like reaction was more suitable to simulate phase I biotransformation processes when compared to the EC system. Using cyclic convolution (CV) and square wave voltammetry as well as controlled potential electrolysis led to the oxidation of moniliformin as described by Toro et al. [121]. The complex electrooxidation mechanism led to reaction products by decomposition of moniliformin but further structural elucidation was not performed. In 2015 Nieto et al. [122] investigated the electrochemical reduction of sterigmatocystin, a mycotoxin, which is a precursor of the genotoxic aflatoxin B1. Toro et al. [121] used a glassy carbon working electrode and observed the irreversible dimerization of moniliformin. Possible chemical structures of the dimeric species were proposed on results based on theoretical calculations. 

Taken together, only a few studies are available yet describing alternative simulation methods like electrochemistry or Fenton-like reaction. However, due to an increasing concern about mycotoxins and their related TPs the trend of using new simulation methods in Mycotoxin Res. is ongoing. When discussing the results of a transformation study, however, it should always be kept in mind that the findings may vary depending on the simulation method used.

## 4. Conclusions and Perspectives

The current state of simulating TPs was reviewed with emphasis on selected classes of toxicological relevant compounds: pesticides, drugs, brominated flame retardants, and mycotoxins. According to the main transformation sources and forces (photo-, bio-, and chemical oxidation) efficient simulation methods were developed. However, simulation methods are generally not able to selectively reproduce the variety of natural transformation processes. It should be still considered that electro- or photochemical simulation methods do not only generate selective transformation products because of diffuse impact of energy to the molecules. Consequently, a multitude of TPs is obtained whose relevance for real-life processes should still be evaluated by comparative measurements. However, the simulated TPs suggested the opportunity of elucidating unknown biotransformation or degradation processes. It should be noted that degradation experiments were often carried out by photochemical experiments and metabolism studies by in vitro or in vivo experiments. Electrochemistry is mostly used for metabolism experiments so far, but first degradation products are also investigated. The general use of all available simulation methods should be more focused, independent of the research area and compound class. 

For the future, research on TPs of toxicological organic compounds will continuously gain relevance. They could represent risks for environmental matrices and food due to possibly adverse effects of their still unknown properties. Simulation methods can help not only to study transformation processes and to identify new TPs but also to produce TPs in larger quantities for subsequent investigations (e.g., toxicity or real-life screening). 

Electrochemistry (EC) has become one of the most important method for the simulation of transformation products (TPs), underpinned by the rapidly increasing number of publications in recent years. Oxidation and reduction processes can be studied very simple including the variation of experimental parameters, that help to adjust real-life conditions. EC can be used as simulation method for biotransformation processes as well as for environmental processes. The selectivity of generation of specific TPs must be improved. Furthermore, the online coupling of EC to (high-resolution) mass spectrometry ((HR)MS) facilitates the unambiguous identification of derived TPs. The broad applicability including a TP synthesis unit will increase the importance of EC-(HR)MS as simulation method for toxicological relevant compounds in the future.

## Figures and Tables

**Table 1:** Selected pesticides and their transformation products (TPs) in different prediction methods.

Name of Compound/s	Type of Pesticides	Matrices	TPs Prediction/Authentication Methods	Simulated Transformation Mechanisms	Ref
Azoxystrobin Difenoconazole Thiacloprid	fungicide fungicide insecticide	brassica species vegetables (pak choi and broccoli)	Suspect screening, in silico fragmentation and authentic standards	Hydrolysis, hydroxylation, dealkylation (*O*-, *N*- & *S*-), dehydrogenation, glucuronidation, esterification	[47]
Chlorpyrifos	insecticide	fruits and spices	Synthesis by EC and UV and used for targeted screening	*P*-oxidation, *O*-dealkylation, hydrolysis, −Cl +H	[48]
Ethoxyquin	growth regulator	fish feed	Synthesis by chemical (H_2_O_2_ and DPPH) and used for authentication	*N*-oxide, hydroxylation, *O*-dealkylation, dimerization, epoxidation, quinone formation	[51]
Forchlorfenuron	growth regulator	kiwifruit	Comparison with authentic standards	+OH, −Cl +H, *N*-dealkylation, −Cl +OH, glucuronidation	[52]
Isoproturon	herbicide	plant leaf	UV and sunlight (untargeted)	Hydroxylation, *N*-dealkylation	[53]
Bentazon	herbicide	-	Photocatalysis (TiO_2_, ZnO)	*N*-dealkylation, hydroxylation, dimerization	[57]
Cyprodinil	fungicide	-	UV–Vis	Isomerization, cyclization, hydroxylation	[58]
Methyl parathion	insecticide	-	MnO_2_ (chemical)	*P*-oxidation, hydrolysis, dealkylation	[59]
Fluopyram	herbicide	-	UV and sunlight (untargeted)	−Cl +H, −Cl +OH, cyclization via −HCl	[60]
Fluopyram	herbicide	commercially available microsomes	Liver microsomes and EC	Hydroxylation, *N*-dealkylation, −Cl +H, −Cl +OH, dehydrogenation, epoxidation	[62]
Chlorpyrifos	insecticide	commercially available microsomes	Liver microsomes and EC	*P*-oxidation, *O*-dealkylation, hydrolysis, −Cl +H	[63]
Metribuzin	herbicide	tomato	Comparison with authentic standards	Deamination, oxidative desulfuration	[65]
Flonicamid	insecticide	orange	Comparison with authentic standards	Hydrolysis of amides, deamination by hydrolysis	[66]
Quinoxyfen	fungicide	water	UV and untargeted	−Cl +OH, hydroxylation, cyclization (−H_2_O, −HCl), hydrolysis	[67]
DDT and DDT-related cpds	insecticide	dolphin serum	Comparison with authentic standards	Olefin via −HCl, −Cl +OH	[68]

**Table 2:** Selected studies of drugs and their TPs using different simulation methods.

Drugs	Group of Drugs	TPs Prediction/Authentication Methods	Simulated Transformation Mechanisms *	Toxicity Test of TP	Degra Dation Kinetic	Ref
Amoxicillin, Cefotaxime, Linezolid, Moxifloxacin, Metronidazole, Fluconazole	Antibiotic Antifungal	EC-reactor RLM In vivo (urine samples)	Red, Deh, Dealk, OH, X-ox, Dehal, GSH Red, Deh, Dealk, OH, X-ox, Dehal, Red, X-ox, OH, Alkyl			[69]
Acebutolol, Atenolol, Propranolol, Pindolol, Oxprenolol, Cicloprolol, Pirbuterol, Mexiletine, Propafenone	β-blocker β2-adrenoceptor Antiarrhythmic	EC-reactor In vivo (urine and plasma samples)	OH, X-ox, Dealk, Alkyl, Red, GSH OH, X-ox, Dealk			[72]
Buspirone Promazine Testosterone 7-ethoxycoumarin	Anxiolytic Antipsychotics Anabolic steroid Test substance (CYP450)	HLM Photocatalytic (TiO_2_) EC-Fenton EC-Reactor	OH, Deh, X-ox, Dealk, Hyd, OH, Deh, X-ox, Dealk, Hyd OH, Deh, X-ox, Hyd Deh, X-ox, Dealk			[26]
Oxacillin	β-lactam antibiotic	Photocatalytic (TiO_2_) Sonochemisty EC-reactor Photo-Fenton	X, ox, rCleav, OH X-ox, rCleav X-ox, rCleav X-ox, rCleav, OH			[73]
Trimethoprim	Anti-infective	Electrolysis EC-Fenton EC reactor	OH OH, Dealk OH			[18]
Moclobemide, Toloxatone	Antidepressant	Photocatalytic (TiO_2_) HLM	X-ox, OH, Dealk, Deh, Dehal, rCleav X-ox, OH, Dealk, Deh		X	[74]
Clozapine	Antipsychotic	Photocatalytic (TiO_2_, ZnO) HLM	X-ox, OH, Hyd, Dealk, Dehal, rCleav X-ox, OH, Hyd, Dealk, Dehal, rCleav		X	[70]
Tiapride	Antipsychotic	Photolytic Photocatalytic (TiO_2_, H_2_O_2_)	Dealk, OH, Red, Desulf, X-ox Dealk, OH, Red, Desulf, X-ox, rCleav, Deh	X	X	[75]
Propranolol	β-blocker	Photolysis	OH, rCleav, X-ox, Ox	X	X	[27]
Vincristine	Anticancer	Aerobic activated sludge	rCleav, Ox, Deca, Dealk, Deh,		X	[76]
Venlafaxine	Antidepressant	Photocatalytic (UV/TiO_2_)	OH, Alkyl, Deh, X-ox,	X	X	[77]
Triclosan	Antimicrobial	Electrochemical reactor	OH, Ox, Dehal, Alky	X		[78]
Ibuprofen	Nonsteroidal anti-inflammatory	UV/chlorine UV/H_2_O_2_	OH, Decar, Dealk, +Cl, rCleav	X	X	[79]
Omeprazole	Proton pump inhibitors	Photolysis	Red, Dehyd, OH, Ox	X	X	[80]
Ifosamine, Cyclophosphamide	Cytostatic	Photocatalytic (TiO_2_, Pt-TiO_2_)	OH, Dealk, Ox		X	[81]
Amoxicillin, Ampicillin	Antibiotic	Photolysis	OH, Deh, Dealk		X	[82]
Carbamazepine	Antiepileptic	UV/chlorine	Red, OH, +Cl, Hyd, Deh	X	X	[83]
Naproxen	Nonsteroidal anti-inflammatory	UV and solar Photolysis	Dealk, Hydr, OH	X	X	[84]

Red: reduction (−O), Deh: Dehydration (−H), Dealk: Dealkylation (−C_x_H_y_), OH: Hydroxylation (+OH, −H), X-ox: Oxidation heteroatom (N, S), Ox: Oxidation (+O), Hyd: Hydrogenation (+H), Dehal: Dehalogenation, rCleav: ring cleavage opening, Decar: Decarboxylation (−CO_2_), Desulf: Desulfonation (−S), Alkyl: Alkylation (+C_x_H_y_). Studies were toxicity tests or the degradation kinetic is determined are marked with X. * Further reactions like isomerization, cyclization, and breakdown to small fragments are not mentioned.
